# Identifying Health-Related Discussions of Cannabis Use on Twitter by Using a Medical Dictionary: Content Analysis of Tweets

**DOI:** 10.2196/35027

**Published:** 2022-02-25

**Authors:** Jon-Patrick Allem, Anuja Majmundar, Allison Dormanesh, Scott I Donaldson

**Affiliations:** 1 Department of Population and Public Health Sciences Keck School of Medicine University of Southern California Los Angeles, CA United States; 2 Department of Surveillance and Health Equity Science American Cancer Society Kennesaw, GA United States

**Keywords:** cannabis, marijuana, Twitter, social media, adverse event, cannabis safety, dictionary, rule-based classifier, medical, health-related, conversation, codebook

## Abstract

**Background:**

The cannabis product and regulatory landscape is changing in the United States. Against the backdrop of these changes, there have been increasing reports on health-related motives for cannabis use and adverse events from its use. The use of social media data in monitoring cannabis-related health conversations may be useful to state- and federal-level regulatory agencies as they grapple with identifying cannabis safety signals in a comprehensive and scalable fashion.

**Objective:**

This study attempted to determine the extent to which a medical dictionary—the Unified Medical Language System Consumer Health Vocabulary—could identify cannabis-related motivations for use and health consequences of cannabis use based on Twitter posts in 2020.

**Methods:**

Twitter posts containing cannabis-related terms were obtained from January 1 to August 31, 2020. Each post from the sample (N=353,353) was classified into at least 1 of 17 a priori categories of common health-related topics by using a rule-based classifier. Each category was defined by the terms in the medical dictionary. A subsample of posts (n=1092) was then manually annotated to help validate the rule-based classifier and determine if each post pertained to health-related motivations for cannabis use, perceived adverse health effects from its use, or neither.

**Results:**

The validation process indicated that the medical dictionary could identify health-related conversations in 31.2% (341/1092) of posts. Specifically, 20.4% (223/1092) of posts were accurately identified as posts related to a health-related motivation for cannabis use, while 10.8% (118/1092) of posts were accurately identified as posts related to a health-related consequence from cannabis use. The health-related conversations about cannabis use included those about issues with the respiratory system, stress to the immune system, and gastrointestinal issues, among others.

**Conclusions:**

The mining of social media data may prove helpful in improving the surveillance of cannabis products and their adverse health effects. However, future research needs to develop and validate a dictionary and codebook that capture cannabis use–specific health conversations on Twitter.

## Introduction

The cannabis product and regulatory landscape is changing in the United States. A total of 34 states have legalized medical cannabis, and 10 states have legalized cannabis for adult recreational use (ie, for people aged 21 years or older) [1]. Against the backdrop of these changes, there have been increasing reports on health-related motives for cannabis use [2,3] and adverse events from its use [4]. Examples of motivations for cannabis use include treatment for clinical health conditions (eg, glaucoma, nausea, AIDS-associated anorexia, epilepsy, multiple sclerosis, and chronic pain) [5,6]—a use supported by the US Food and Drug Administration (FDA). Additionally, studies have shown that motivations for cannabis use have been based on the perceived benefits of its use, including its use as a sleep aid [2] and an aid for coping with stress or anxiety [3]. The low perception of harm from cannabis use when compared to that from other psychoactive drugs has also been documented as a motivation for its use [7]. However, cannabis use has been associated with adverse events, such as impaired short-term memory, impaired motor coordination, paranoia, and psychosis [6]; increased levels of depression and anxiety over time; symptoms of chronic bronchitis; addiction; and altered brain development [3,5,6]. Although the literature on the motivations for and effects of cannabis use is developing, medical experts recommend establishing a centralized federal agency for reporting, researching, and regulating cannabis products as a timely public health surveillance strategy [4]. The surveillance of the adverse health effects of cannabis is also a key priority of the US FDA [8]. The FDA’s MedWatch program conducts the surveillance of serious adverse effects from cannabis use, but doubts have been raised over how effective this surveillance system is in identifying reports of cannabis safety signals [9].

The surveillance of health-related behaviors includes the use of digital data sources [10]. Publicly accessible data from individuals who post to social media platforms, such as Twitter, have been used to capture and describe the context of cannabis use [11,12]. However, health-related conversations surrounding its use have been understudied, and there has been a lack of cannabis-related studies that use social media data. The mining of social media data permits the collection and analysis of qualitative information, is noninvasive (ie, no demand effect), minimizes recall error, and allows for data to be captured in real time. Twitter has been a growing tool in health research, and it has been used for various purposes, including content analysis, surveillance, recruitment, intervention, and network analysis [13]. Twitter in particular reflects the views, attitudes, and behaviors of millions of people and is used by 22% of US adults (24% of men, 21% of women, 21% of White Americans, 24% of African Americans, and 25% of Hispanic Americans), with 42% of individuals using the platform daily [14].

This study attempted to determine the extent to which a medical dictionary—the Unified Medical Language System Consumer Health Vocabulary (CHV) [15]—could accurately identify cannabis-related motivations for use and health consequences of cannabis use based on Twitter posts in 2020. The findings may be useful to state- and federal-level regulatory agencies as they grapple with identifying cannabis safety signals in a comprehensive and scalable way.

## Methods

### Study Design

Twitter posts containing the cannabis-related terms *blunt*, *bong*, *budder*, *cannabis*, *cbd*, *ganja*, *hash*, *hemp*, *indica*, *kush*, *marijuana*, *marihuana*, *reefer*, *sativa*, *thc*, and *weed* were obtained from January 1 to August 31, 2020. These terms were informed by prior research that focused on comprehensively collecting cannabis-related posts on Twitter [11]. To treat each observation as independent, retweets were removed, leaving a total of 16,703,751 unique posts that contained these terms during this time. We used the following two dictionaries: (1) the Unified Medical Language System CHV [15], which comprises 13,479 medical terms (symptoms and diseases) that are used by consumers and health care professionals to describe health conditions, and (2) a list of 177 colloquial terms that were generated collaboratively by 2 trained coders and were related to the CHV terms when pertinent (eg, the colloquial expression of *inebriation* is *drunk*). The CHV has been used in prior research for the surveillance of health discussions about e-cigarette use or vaping on Twitter [16]. CHV terms are available at no cost to applicants who have a license, which is assigned upon the completion of a web-based application process. A sample of 609,227 cannabis-related posts referenced at least 1 of these terms.

We then identified and removed posts from social bots (ie, automated Twitter accounts) to reliably describe the public’s health-related motivations for cannabis use or the perceived health effects of its use [17]. In order to distinguish nonbots from social bots, we relied upon Botometer (Observatory on Social Media) [18,19]. This program analyzes the features of a Twitter account and provides a score based on how likely the account is to be a social bot. The Botometer threshold was set to ≥4 on an English rating scale of 1 to 5. All Twitter accounts were screened after data were collected (ie, not in real time). During this process, 127,140 accounts responsible for the tweets in our data were deleted from Twitter. As a result, these accounts could not be processed through Botometer, and their posts were removed from our data. Of the 261,134 available accounts, 15,245 were marked as bots and removed. The final analytic sample contained 353,353 posts from 245,889 unique nonbot accounts.

Each post from the final sample was classified into at least 1 of 17 a priori health-related categories [16] by using a rule-based classifier. Each category was defined by the terms in the two dictionaries. The 17 health-related categories included 14 categories from prior research [16] and 3 additional categories that were unique to this study, accounting for the potential psychoactive effects of cannabis use (the “Cognitive” category), topical cannabis products (the “Dermatological” category), and the intersection of cannabis and food additives (the “Poisoning” category). A post could belong to multiple categories. The 17 categories, example keywords, and prevalence of keywords from each category can be found in Table 1.

A stratified random sample of posts (n=1092) was extracted from the corpus (n=353,353) based on the original classifications of the posts by using the rule-based classifier. A coding procedure (Multimedia Appendix 1 contains the complete codebook) was used to determine if each post pertained to a health-related motivation for cannabis use, a perceived adverse health effect of cannabis use, or neither. Two trained coders double coded each post independently, with κ values ranging from 0.790 to 0.856. Discrepancies were resolved by the two coders and the first author. This analysis served as a validation procedure for the rule-based classifier.

**Table 1:** Health categories, example keywords, and the frequency of occurrence on Twitter (N=353,353).

Health categories	Example keywords	Frequency, n (%)
Cancer	*Cancer*, *tumor*, and *malignant*	13,834 (3.92)
Cardiovascular	*Stroke*, *heart attack*, and *blood pressure*	1810 (0.52)
Cognitive	*Unconscious* and *attention*	8807 (2.49)
Death	*Die*, *kill*, and *lost life*	31,590 (8.95)
Dermatological	*Itchy*, *acne*, and *blister*	1557 (0.44)
Gastrointestinal	*Belly*, *belch*, *vomit*, and *puke*	10,434 (2.95)
Immune System	*Flu*, *common cold*, and *allergy*	12,229 (3.46)
Injury	*Injury*, *rupture*, *wound*, and *bruise*	19,490 (5.52)
Mental health	*PTSD*, *ADHD*, and *jittery*	100,155 (28.34)
Neurological	*Coma*, *dizzy*, and *lightheaded*	56,347 (15.95)
Other	*Anemia*, *jaundice*, and *mumps*	44,111 (12.48)
Pain	*Painful*, *achy*, and *cramping*	38,335 (10.85)
Poisoning	*Toxic*, *poisonous*, and *noxious*	8345 (2.36)
Pregnancy or in utero	*Pregnant*, *preggers*, and *miscarriage*	4760 (1.35)
Respiratory	*Cough*, *wheeze*, and *black lung*	16,616 (4.70)
Stress	*Stressed* and *cortisol*	13,372 (3.78)
Weight	*Fat*, *obese*, *weight*, and *stoutness*	5888 (1.67)

### Ethical Considerations

All analyses relied on public, anonymized data; adhered to the terms and conditions, terms of use, and privacy policies of Twitter; and were performed under institutional review board approval from the authors’ university. To protect privacy, no tweets were reported verbatim in this report.

## Results

The validation process indicated that the medical dictionary could identify health-related conversations in 31.2% (341/1092) of posts (Table 2). Specifically, 20.4% (223/1092) of posts were identified as posts related to a health-related motivation for cannabis use, while 10.8% (118/1092) of posts were identified as posts related to a health-related consequence from cannabis use. The health-related conversations about cannabis use included those about issues with the respiratory system, stress to the immune system, and gastrointestinal issues, among others.

**Table 2:** The validation results for the rule-based classifier.^a^

Category			Motivations, n (%)		Consequence, n (%)		Neither, n (%)		Total^b^, n
**Medical term**									
	*Cancer*	15 (42.9)		4 (11.4)		16 (45.7)		35	
	*Cardiovascular*	1 (20)		1 (20)		3 (60)		5	
	*Cognitive*	5 (18.5)		3 (11.2)		19 (70.3)		27	
	*Death*	4 (4)		7 (8)		79 (88)		90	
	*Dermatological*	0 (0)		0 (0)		4 (100)		4	
	*Gastrointestinal*	6 (21)		1 (3)		22 (76)		29	
	*Immune system*	2 (6)		2 (6)		31 (88)		35	
	*Injury*	1 (2)		5 (9)		49 (89)		55	
	*Mental health*	89 (31.8)		19 (6.7)		172 (61.4)		280	
	*Neurological*	18 (11.3)		40 (25)		102 (63.7)		160	
	*Other*	33 (26.6)		7 (5.6)		84 (67.8)		124	
	*Pain*	28 (25.7)		3 (2.8)		78 (71.5)		109	
	*Poison*	2 (8.3)		6 (25)		16 (66.7)		24	
	*Pregnant*	2 (14.3)		2 (14.3)		10 (71.4)		14	
	*Respiratory*	0 (0)		17 (36.2)		30 (63.8)		47	
	*Stress*	17 (44.7)		1 (2.6)		20 (52.7)		38	
	*Weight*	0 (0)		0 (0)		16 (100)		16	
Total^c^			223 (20.4)		118 (10.8)		751 (68.8)		1092^d^

^a^The values in the *Motivations*, *Consequence*, and *Neither* columns show the number and percentage of posts related to health-related motivations for cannabis use, health-related consequences from cannabis use, or neither, respectively, for each medical term.

^b^The *Total* column refers to the total number of tweets coded per medical term.

^c^The values in the *Total* row show the number and percentage of posts related to health-related motivations for cannabis use, health-related consequences from cannabis use, or neither, respectively, for all medical terms.

^d^The total number of tweets in the subgroup.

## Discussion

### Principal Findings

This study determined the extent to which a commonly used medical dictionary of health effects could accurately identify cannabis-related motivations for use and health consequences of cannabis use based on Twitter posts in 2020. This is the first study to date to use a high-quality medical dictionary of consumer-oriented health terms to capture the public’s expressions of health concepts and thereby identify health conversations about cannabis use. The findings suggest that a medical dictionary alone is limited in its ability to identify health-related conversations in a cannabis context. The posts discussed the respiratory system, stress to the immune system, and gastrointestinal problems. The posts also discussed mental health, pain, injuries, and poisonings, among other potential health effects.

Previous research has identified motivations for cannabis use, including using cannabis to treat chronic conditions (eg, glaucoma, nausea, AIDS-associated anorexia, epilepsy, multiple sclerosis, and chronic pain) [2,5,6], using it as a sleep aid [2], and using it to help improve mental health (eg, stress, anxiety, and depression) [3]. Previous research has also identified adverse reactions associated with cannabis consumption based on search engine queries and found that such queries revealed many of the known adverse effects of cannabis use, such as coughing and psychotic symptoms, as well as plausible reactions that could be attributed to cannabis use, such as pyrexia [20]. A prior content analysis of 5000 tweets about “dabbing” (the use of a high-potency cannabis-related product) from a 30-day period in 2015 showed that the most common physiologic effects from this form of cannabis use were the loss of consciousness and respiratory effects, such as coughing [21]. Our study compliments prior research by using a professionally used term dictionary. It also indicates that the public made varied health-related references in their conversations about cannabis on Twitter. However, if the mining of social media data is to be proven helpful in the surveillance of cannabis products and their adverse health effects, the use of a standardized medical term dictionary alone will not suffice in the identification of cannabis safety signals. Future research will need to develop a codebook and term dictionary that incorporate a priori categories and data-driven inductive approaches that capture nuanced cannabis and health-related conversations on Twitter.

### Limitations

This study focused on posts to Twitter, and the findings may not extend to other social media platforms. Additionally, the posts in this study were collected from an 8-month period in 2020; thus, the findings may not extend to other time periods. The data collection process relied on Twitter’s Streaming application programming interface, which prevented the collection of posts from private accounts. As such, the findings may not generalize to all Twitter users or to the US population. The people responsible for each post in this study were not examined, and as a result, we could not describe the demographics of the Twitter users in this study. Further, Twitter posts can contain misspellings, and our lexicon-based exact matching approach likely missed these expressions. The CHV has also not been updated since 2011, which may in part explain its limited ability to identify health-related conversations in a cannabis context. Finally, this study could not determine modes of cannabis use or whether cannabis use was coupled with other substances or medications, which may impact perceived health effects.

### Conclusions

Medical experts and regulatory agencies have called for the improved surveillance of cannabis products and the adverse health effects from cannabis use. Until the limitations with syndromic surveillance and hospital data systems for cannabis (eg, accessibility of data and timeliness) are resolved, the mining of social media data may clarify the public’s experiences with cannabis use. The development of a validated dictionary and codebook that capture cannabis-specific health conversations may be key to advancing future efforts in the surveillance of Twitter data. A robust, national-level surveillance system for cannabis-related health effects may benefit from using real-time social media surveillance data on health effects and should consider using data from other sources (eg, emergency room visits and survey data).

## Appendix

Multimedia Appendix 1 Codebook for monitoring health-related discussions about cannabis use on Twitter.

## Abbreviations

CHV: Consumer Health Vocabulary
FDA: Food and Drug Administration
