# Supplementary material for: Identifying Health-Related Discussions of Cannabis Use on Twitter by Using a Medical Dictionary: Content Analysis of Tweets
Source: JMIR Form Res. 2022 Feb 25;6(2):e35027. doi: 10.2196/35027 (PMC8917433; doi:10.2196/35027)
Supplement: Multimedia Appendix 1 [file formative_v6i2e35027_app1.docx]

Monitoring Health-Related Discussions about Cannabis Use on twitter

***Unit of Analysis:*** Read the content of the tweet. If a theme is present in the post (text) place a “1” in the motivation, consequence, or neither cell. If a theme is not present, please place a “0”. Each post can contain more than one theme.

## Codebook

| Medical Term | Motivation for  Cannabis Use | Consequence of Cannabis Use |
| --- | --- | --- |
| Neurological | Posts say that cannabis can restore or enhance brain functioning, such as relieving drowsiness, lightheadedness, or treating other neurological issues like epilepsy. | Posts say that cannabis disrupts normal brain function, resulting in difficulty staying awake, feeling dizzy, or feeling knocked out |
|  | Example: ‘The GP for a seven-year-old boy whose mother uses #cannabis-based drugs to treat his epilepsy has agreed to write to the Scottish Government in an attempt to secure him a prescription.’ | Example:‘not even haha this is funny amusing but like feel dizzy for four hours and throw up bad.’ |
| Mental health | Posts say that cannabis can make you feel calm and/or relieve mental health issues, including feelings of sadness, trauma, or breakdowns. The post may also say that cannabis can help manage the symptoms of mental health disorders like PTSD, ADHD, or anxiety. | Posts describe negative mental health conditions worsened by cannabis, such as being moody, nervous, paranoid, or tense. |
|  | Example: ‘@bitandbang Real talk I swapped out ADHD meds with CBD pills and have been living a way happier and healthier life ever since! Happy 420.’ | Example: ‘This suggests that marijuana use has a significant impact on interpersonal behaviors, of which users are not aware. Additional research has found social-emotional deficits in marijuana users, and increases in hostility or aggression.’ |
| Death | Posts claim that cannabis is less likely to cause death compared to other substances such as alcohol or opioids. | Posts claim that cannabis use lead to a near-death experience, like almost dying or losing consciousness from cannabis use. |
|  | Example: ‘More than 150,000 Americans die of alcohol abuse each year. But in more than 10,000 years of usage, no one has ever died from marijuana...’ | Example: ‘That first bong rip have you feeling like death is imminent’ |
| Injury | Posts mentions cannabis as a way to heal an injury, swelling, or other damage to the body. Posts may also speak about healing ‘injured’ emotions or feelings with cannabis. | Posts describe an injury as a result of using cannabis products (e.g., burned finger). |
|  | Example: ‘alcohol burns like a bitch but cuts the time in half. And then put hemp oil on it to heal.’ | Example: ‘They are made for smoking roaches (when the blunt or joint gets so small it starts burning your fingers) lol.’ |
| Respiratory | Posts say cannabis can help manage or relieve breathing issues, such as cough, shortness of breath, or panting. | Posts suggest that cannabis use triggered a cough, phlegm, feeling out of breath, or other chest problem. |
|  | Example: ‘yes i have an upper respiratory infection. yes i still want to smoke a blunt. we exist.’ | Example: ‘Hate having a residual weed cough’ |
| Pain | Posts claim that cannabis can help manageor treat pain, especially compared to painkillers, or for other pain issues like headache, backpain, fibromyalgia, etc. | Posts describe a pain condition that was triggered by or worsened by cannabis use (e.g., toothache, earache, migraine). |
|  | Example: ‘I cant do flower (im allergic) but the edibles are my savior when it comes to pain management. Absolutely safer than pharmaceutical garbage. Cbd is my savior especially after my summer of multiple surgeries.’ | Example: ‘I have a medical condition and refuse weed for medicinal purposes. The smell makes my seizures worse and my migraines just as bad. Did the Governor think about those of us who can't/won't use the weed because it makes us more sick? Of course not.’ |
| Cancer | Posts say that cannabis may cure some forms of cancer, like Hodgkinson’s, sarcoma, leukemia, etc. | Posts say cannabis may cause cancer. |
|  | Example: ‘I swear one day we will find a cannabis or hemp variant that melts tumors instantly. This sacred plant has so much more potential, gut feeling cancer will end with cannabis.’ | Example: ‘bruh went from sugar causes cancer to that synthetic weed y'all smoke causes cancer too’ |
| Gastrointestinal | Posts suggest cannabis can relieve stomach issues, including bloating, constipation, issues with appetite, and cramps. | Post says that cannabis may affect stomach problems, such as diarrhea or indigestion. |
|  | Example: ‘Facts. Drinking makes my stomach hurt. Weed makes my stomach stop hurting.’ | Example: ‘available data raise serious concerns about potential harm from #CBD, diarrhea and changes in mood’ |
| Cardiovascular | Posts suggest that cannabis may prevent heart attack, stroke, heat flash, or other cardiovascular complications. | Posts describe heart problems from cannabis use like stroke, heart palpitations, or elevated cholesterol. |
|  | Example: ‘You are welcome my friend. There are so many articles and studies demonstrating the benefits of cannabis use for stroke. These were two that I had handy. This is truly god’s plant. Nature at its best.’ | Example: ‘Made a bong out of a plastic water bottle, thinking I’m cute, took one too many hits, everything was in slow motion, I drank 3 bottles of water, thought I was having a stroke and ended in the hospital and they literally said‚ sleep it off you’ll be fine‚ and charged me $140’ |
| Weight | Posts claim that cannabis can be used to lose weight and manage weight-related problems like obesity. | **Posts suggest cannabis may cause weight gain, obesity, or other weight-related issues.** |
|  | Example: ‘Evidence shows that cannabis users are much less likely to develop metabolic syndrome, a significant risk factor for obesity.’ | **Example: ‘Hope some of y’all put that weed down and go to the gym to lose weight.’** |
| Stress | Post suggests that cannabis may help with stress-related illnesses, such as Cushing’s syndrome, hypercortisolism, or other stress issues, like irritability and shaking. | Posts suggest cannabis use may trigger stress. |
|  | Example: ‘#CBD appears to have a positive interaction with serotonin receptors in the brain. #Serotonin impacts a range of functions in the body, #anxiety, stress & depression including a person’s emotional state’ | Example: ‘If your immediate response to stress is rolling up and smoking weed then ur addicted. There’s different forms of addiction and that’s one of them.’ |
| Immune System | Post reflects the position that cannabis can treat or alleviate the symptoms of the common cold, allergies, gluten intolerance, etc. | Post reflects the position that cannabis may cause or aggravate symptoms related to the common cold, allergic diseases, gluten intolerance, etc. |
|  | Example: ‘It helps me when I feel nauseous, or if I actually get sick I smoke after & it makes me feel better.’ | Example: ‘I really don't think anyone's ever died from smoking weed unless they were highly allergic.’ |
| Pregnancy/In-utero | Posts suggest that cannabis can help with undesirable health issues during pregnancy (e.g., morning sickness). | Posts suggest that cannabis may lead to complications during pregnancy such as birth defects, or discontinuing cannabis use during pregnancy may create negative health effects like withdrawals. |
|  | Example: ‘Oh, I gotcha...I smoked weed my entire pregnancy with my son because I couldn’t ever eat and I was constantly having morning sickness...but maybe CBD might help? they even have candy so you don’t have to smoke’ | Example: ‘These compounds in THC and CBD induce birth defects that are very similar to what we see in fetal alcohol syndrome. - Scott Parnell, PhD, at @UNC_SOM’ |
| Cognitive | Posts suggest that cannabis may improve cognitive performance, such as increased focus, concentration, and attention | Post suggests that cannabis may negatively influence cognitive performance, such as poor concentration or other outcomes like dropping out of school. |
|  | Example: ‘Weed doesn’t demotivate people or make them perform less.’ | Example: ‘Don't get busted with weed in college and drop out because Uncle Sam won't pay my degenerate ass to study.’ |
| Dermatological | Posts mention a range of skin benefits and/or alleviation of pre-existing skin issues related to cannabis use, such as soothing sores, rashes, pale skin, eczema, or dry skin. | Post describes skin -related issues from cannabis use, such as a blemish, crawling skin, dermatitis, ichtyosis, or cellulitis. |
|  | Example: ‘Pain relief with THC induced rub for comfort & getting back to life. Induced with #cannabis & calendula oils for external use helps soothe sore, inflamed & itchy skin conditions. Calendula oil massage also assists in soothing, & softening skin’ | |
| Poisoning | Posts suggest that cannabis may be used to remove poisonous or other harmful toxins from the body.. | Post suggests that cannabis may cause a toxic reaction. |
|  | Example: ‘Cannabis cleans up nuclear radiation and toxic soil.’ | Example: ‘we are going backwards in human health to free unregulated use of public cannabis. The toxic effect on lungs is real, among other side effects.’ |
| Other | Posts suggest that cannabis can improve other health conditions, such as conjunctivitis, hyperglycemia, hypothermia, jaundice, measles, among others. | Posts suggest that cannabis can worsen other health conditions, including anemia, inflammation, glaucoma, gout, and tonsillitis |
|  | Example: ‘CBD oils work for arthritis like Osteoarthiritis and Costochondritis ? As far I know it's good for Parkinson’ | Example: ‘Pain after consuming cannabis, you may be experiencing a reaction to other medication, or an allergic reaction causing inflammation.' |
